# Enzymatic Production of Chondroitin Oligosaccharides and Its Sulfate Derivatives

**DOI:** 10.3389/fbioe.2022.951740

**Published:** 2022-07-13

**Authors:** Weijiao Zhang, Ruirui Xu, Xuerong Jin, Yang Wang, Litao Hu, Tianmeng Zhang, Guocheng Du, Zhen Kang

**Affiliations:** ^1^ The Key Laboratory of Carbohydrate Chemistry and Biotechnology, Ministry of Education, School of Biotechnology, Jiangnan University, Wuxi, China; ^2^ The Science Center for Future Foods, Jiangnan University, Wuxi, China; ^3^ The Key Laboratory of Industrial Biotechnology, Ministry of Education, School of Biotechnology, Jiangnan University, Wuxi, China

**Keywords:** chondroitin, oligosaccharides, chondroitin sulfate, chondroitinase ABC I, sulfation system

## Abstract

Chondroitin sulfate (CS) has a wide range of physiological functions and clinical applications. However, the biosynthesis of chondroitin oligosaccharides (o-CHs) and sulfate derivatives with specific length is always challenging. Herein, we report enzymatic strategies for producing homogeneous o-CHs and its sulfate derivatives from microbial sourced chondroitin. Chondroitin disaccharides, tetrasaccharides, hexasaccharides, octasaccharides, and decasaccharides with defined structure were produced by controllably depolymerizing microbial sourced chondroitin with an engineered chondroitinase ABC I. The highest conversion rates of the above corresponding o-CHs were 65.5%, 32.1%, 12.7%, 7.2%, and 16.3%, respectively. A new efficient enzymatic sulfation system that directly initiates from adenosine 5′-triphosphate (ATP) and sulfate was developed and improved the sulfation of chondroitin from 8.3% to 85.8% by optimizing the temperature, sulfate and ATP concentration. o-CHs decasaccharide, octasaccharide, hexasaccharide, tetrasaccharide and disaccharide were modified and the corresponding sulfate derivatives with one sulfate group were prepared. The enzymatic approaches constructed here for preparing o-CHs and its sulfate derivatives pave the way for the study of structure-activity relationship and applications.

## Introduction

Chondroitin sulfate (CS) as one type of glycosaminoglycans is composed of a disaccharide repeating unit (GlcA-β-1,3-GalNAc-β-1,4-, GlcA = glucuronic acid, GalNAc = *N*-acetylgalactosamine) with different sulfation patterns (Kang et al., 2018). To date, CS has been widely used in medical (Xu et al., 2008; Ren et al., 2018; Mahtab et al., 2020) and nutraceutical fields (Restaino et al., 2019; Stellavato et al., 2021) while the precursor chondroitin has also been demonstrated to have great potential applications for anti-inflammatory treatment (Xu et al., 2019; Russo et al., 2020; Vassallo et al., 2021) and enhancing chondrogenic cell proliferation (Stellavato et al., 2016). Recently, many studies have reported that the bioactivities of CS are closely related to its sulfation pattern (Poh et al., 2015; Vessella et al., 2019) and molecular weight (Xiao et al., 2014; Zhao et al., 2021). In particular, CS oligosaccharides with defined length are more conducive to the studies of structure-activity relationship (Shida et al., 2017; Ji et al., 2020). For instance, it has been reported that CS disaccharide displays inhibitory effect on human colon cancer cells *in vitro* (Rani et al., 2019) while CS tetrasaccharide is defined as the smallest motif for promoting neuronal outgrowth *in vitro* (Tully et al., 2004). Meanwhile, nonadecasaccharide with longer length has the potential for preventing organ damage in the treatment of endotoxemia *in vivo* (Li et al., 2020). Thus, development of new approaches for producing chondroitin oligosaccharides (o-CHs) and sulfate derivatives is imperative and attractive.

In the past decades, great efforts have been dedicated to the synthesis of CS oligosaccharides with chemical (Mende et al., 2016; Ramadan et al., 2020; Wang H. et al., 2020; Zhang et al., 2020) and chemical enzymatic (Boltje et al., 2009; Gao et al., 2019; Zhang et al., 2019) methods. As an alternative route, an enzymatic method for synthesizing o-CHs and CS oligosaccharides has been constructed, which depends on the consumption of UDP-GalNAc and UDP-GlcA monomers (Li J. et al., 2017). Given the high price of these UDP-sugars and with the advances of microbial fermentation of chondroitin (Cimini et al., 2013; Restaino et al., 2013; He et al., 2015; Jin et al., 2016; Cimini et al., 2017; Zhang et al., 2018), other enzymatic strategies for biosynthesis of CS polysaccharides from chondroitin have been established (Zhou et al., 2018; Jin et al., 2020) by recruiting chondroitin sulfotransferases and the aryl sulfotransferase IV for 3′-phosphoadenosine-5′-phosphosulfate (PAPS) regeneration (Burkart et al., 2000). However, the requirement of expensive substrate 3′-phosphoadenosine-5′-phosphate (PAP) and the generation of toxic by-product p-nitrophenol (PNP) limit its wide application. Thus, development of new sulfation systems for biosynthesis of oligosaccharides sulfate derivatives from microbial sourced chondroitin is promising especially with the approvement of non-animal CS as food ingredients by the United States Food and Drug Administration (FDA) (Volpi et al., 2019).

In the present study, our aim is to construct green facile enzymatic approaches for producing homogeneous o-CHs and its sulfate derivatives. To this end, the depolymerization process of chondroitin with an engineered chondroitinase ABC I (csABC I) was quantitatively analyzed and optimized for preparing specific o-CHs disaccharides, tetrasaccharides, hexasaccharides, octasaccharides and decasaccharides with high conversion rates. On the basis of the construction of an artificially bifunctional enzyme for converting ATP (adenosine 5′-triphosphate) to PAPS (Xu et al., 2021), a new low-cost sulfation system which directly initiates from ATP and sulfate was constructed and optimized for the enzymatic production of chondroitin sulfate A (CSA) and o-CHs sulfate derivatives.

## Materials and Methods

### Depolymerization of Chondroitin

Chondroitin was obtained by 3-L fed-batch fermentation of recombinant *Bacillus subtilis* as described previously (Zhou et al., 2018). csABC I was prepared (protein concentration 1.7 g/L, purity 91.0%) using the recombinant *Escherichia coli* as described previously (Wang Y. et al., 2020). The depolymerization system was carried out in 20 mM Tris-HCl (pH 7.4) buffer solution, and different amounts of csABC I (100, 200, 400, 600, and 800 U/L) were added to depolymerize chondroitin (15.0 g/L) at 35°C in solution. During the depolymerization of chondroitin, 1 ml sample was taken out of the reaction solution at different depolymerization times (10 min, 30 min, 1, 2, 3, 4, 5, and 6 h), and boiled for 10 min to terminate the reactions. After cooling to room temperature, the sample was centrifuged at 8,609 × g for 10 min to remove the insoluble fractions.

### Weight-Average Molar Mass Determination by Gel Permeation Chromatography-High Performance Liquid Chromatography

Depolymerized samples were analyzed by gel permeation chromatography-high performance liquid chromatography (GPC-HPLC) for determining apparent molecular weight (*M*_w_). The GPC-HPLC was used with an Ultrahydrogel linear column (7.8 mm × 300 mm i.d., Waters), eluted with 100 mM sodium nitrate at 40°C at a flow rate of 0.9 ml/min and a run time of 30 min. Dextran with different apparent *M*_w_ values (purchased from National Institute for Food and Drug Control) were used to establish the calibration curve.

### Preparation of Chondroitin Oligosaccharides

The o-CHs samples were filtered through a 3,000 Da ultrafiltration filter to remove the molecular weight greater than 3,000 Da. The separation and purification of o-CHs were carried out on ÄKTA pure chromatography system (GE, United States), which was equipped with anion exchange chromatography HiTrap 16/10 Q FF (GE, United States) and fraction collector F9-C. The column was equilibrated with buffer A (20 mM phosphate-buffered saline, pH 8), and buffer B (20 mM phosphate-buffered saline contains 140 mM NaCl, pH 8) was used for linear elution at a flow rate of 3 ml/min with 3 column volumes. The eluent was monitored at 232 nm to collect each chromatographic peak fraction. Then, the collected samples were desalted with Superdex 30 Increase 10/300 GL column (GE, United States) and lyophilized to obtain the oligosaccharides. Each oligosaccharide sample (200 mg/L) was tested for nucleic acid (Shim et al., 2010) and protein contamination by ultraviolet absorption spectrum (Li et al., 2017) (scanning range 190–300 nm).

### UPLC-MS Analysis of Oligosaccharide

The UPLC was equipped with an CSH C18 column (1.7 μm, 2.1 × 100 mm; Waters) and a PDA detector (Waters, Inc.) to analyze samples at a flow rate of 0.3 ml/min at 45°C. The sample injection volume was 2 µl. Elution A was acetonitrile, and elution B was H_2_O containing 1% (v/v) formic acid. The gradient elution was performed as follows: 0–4 min, 100%–95% B; 4–6 min, 95–90% B; 6–7 min, 90–60% B; 7–8 min, 60%−20% B; 8–10 min, 20%–0% B; 10–12 min, 0% B.

ESI-MS spectra were obtained in negative ionization mode using MALDI SYNAPT Q-TOF MS (Waters, Inc.). In this ESI-MS run program, the capillary voltage was 3.0 kV, the detection voltage was 2.0 kV, and the cone voltage was 20 V. The source block temperature and desolation temperature were 100 and 400°C, respectively. The desolation gas flow rate and the cone gas flow rate were set to 700 L/h and 500 L/h, respectively. The ESI-MS spectra spanned the mass range of 50–2000 m*/z* at a collision energy (eV) of 6/25 V. Besides, the mass was corrected by flow rate of 30 μl/min with 200 pg/μl of leucine enkephalin, and the corrected mass was 554.2615 Da in negative ion mode.

### Nuclear Magnetic Resonance Analysis

The freeze-dried oligosaccharide sample was dissolved with 500 µl D_2_O (99.9%, Sigma-Aldrich). The solutions were applied to NMR analysis with NMR microtubes (O.D. 5 mm, length 180 mm, Norrell) on a Bruker Advance III spectrometer at 600 MHz. NMR chemical shifts (*δ*) and coupling constants (J) were recorded in ppm and Hz, respectively. Data was processed with the MestReNova software.

### Quantitative Analysis of Chondroitin Oligosaccharides

The depolymerized oligosaccharide mixture was quantitatively analyzed by HPLC on an Agilent 1260 instrument equipped with YMC-Pack Polyamine II column (4 mm × 250 mm, S-5 μm, 12 nm) and UV detector at 30°C. The flow rate of mobile phase (acetonitrile: 100 mM NH_4_H_2_PO_4_ = 1:9, v/v) was 0.5 ml/min.

The pure chondroitin disaccharide (CH2), tetrasaccharide (CH4), hexasaccharide (CH6), octasaccharide (CH8) and decasaccharide (CH10) (0.25, 0.5, 1.0, 1.5, and 2.0 g/L) were used to create the standard curves by in-house standards made of the relationship between oligosaccharide peak area with mass concentration by HPLC. The conversion rate of oligosaccharides refers to the mass concentration percentage of oligosaccharides converted from large *M*_w_ chondroitin substrate.

### Digestion of Chondroitin Tetrasaccharide and Chondroitin Hexasaccharide With csABC I

First, pure CH4 (100 mg/L) and CH6 (100 mg/L) were dissolved in 20 mM Tris-HCl buffer (pH 7.4). Then, the oligosaccharide solution was incubated at 35°C for 12 h under 600 U/L csABC I. After the reaction, the reaction solution was boiled for 10 min to terminate the reaction. The depolymerized product was centrifuged at 8,609 ×g for 10 min, and the supernatant was taken for MS determination.

### Enzymatic Synthesis of Oligosaccharides Sulfate Derivatives

C4ST (chondroitin-4-*O*-sulfotransferase, transfer the sulfation group to position 4) was obtained according to the previous description (Jin et al., 2020). Recombinant *Pichia pastoris* (currently known as *Komagataella phaffii*) α (VAVE)-SUMO-C4ST strain was cultured to secrete C4ST. The protein concentration and the purity of the C4ST were 0.2 g/L and 86.9%, respectively. The ASAK^S5^ (a bifunctional PAPS synthase that converts ATP to PAPS) was prepared according to the previous study (Xu et al., 2021). *In vitro* sulfation of chondroitin was performed in the reaction solvent containing Tris-HCl (50 mM, pH 7.5), ATP (10 mM), MgSO_4_ (20 mM), ASAK^S5^ (0.5 g/L), C4ST (1.0 g/L) and chondroitin (2.0 g/L) for 48 h. To promote efficient chondroitin sulfation, the temperature of the reaction was investigated at of 25°C, 30°C, 35°C, 37°C, and 40°C. On this basis, the concentrations of ATP (5, 10, 15, and 20 mM) and MgSO_4_ (20, 30, 40, 50, and 60 mM) were optimized to further improve the reaction efficiency. 500 mg/L o-CHs (CH2, CH4, CH6, CH8, and CH10) were sulfated *in vitro* for 48 h in the reaction solvent containing Tris-HCl (50 mM, pH 7.5), ATP (10 mM), MgSO_4_ (40 mM), ASAK^S5^ (0.5 g/L) and C4ST (1.0 g/L). In addition, the synthesized CS (15 g/L) was depolymerized with csABC I with 800 U/L for 24 h. The CHS2 was purified with anion exchange chromatography (HiTrap 16/10 Q FF column). The depolymerized product was eluted on ÄKTA pure chromatography system with buffer A (20 mM phosphate-buffered saline, pH 8), and buffer B (20 mM phosphate-buffered saline containing 1000 mM NaCl, pH 8) at a flow rate of 3 ml/min with 8 column volumes.

All reactions were terminated by boiling in water for 10 min. The supernatant was collected by centrifugation at 8,609 × g for 10 min to obtain sulfated products, which were depolymerized into CS disaccharides by chondroitinase ABC (csABC, purchased from sigma) for UPLC-MS (ultra-performance liquid chromatography-mass spectrometry) analysis. The degree of sulfation was calculated by the proportion of sulfated disaccharides.

## Results and Disscussion

### Chondroitin Depolymerization and Oligosaccharides Separation

**FIGURE 1:**
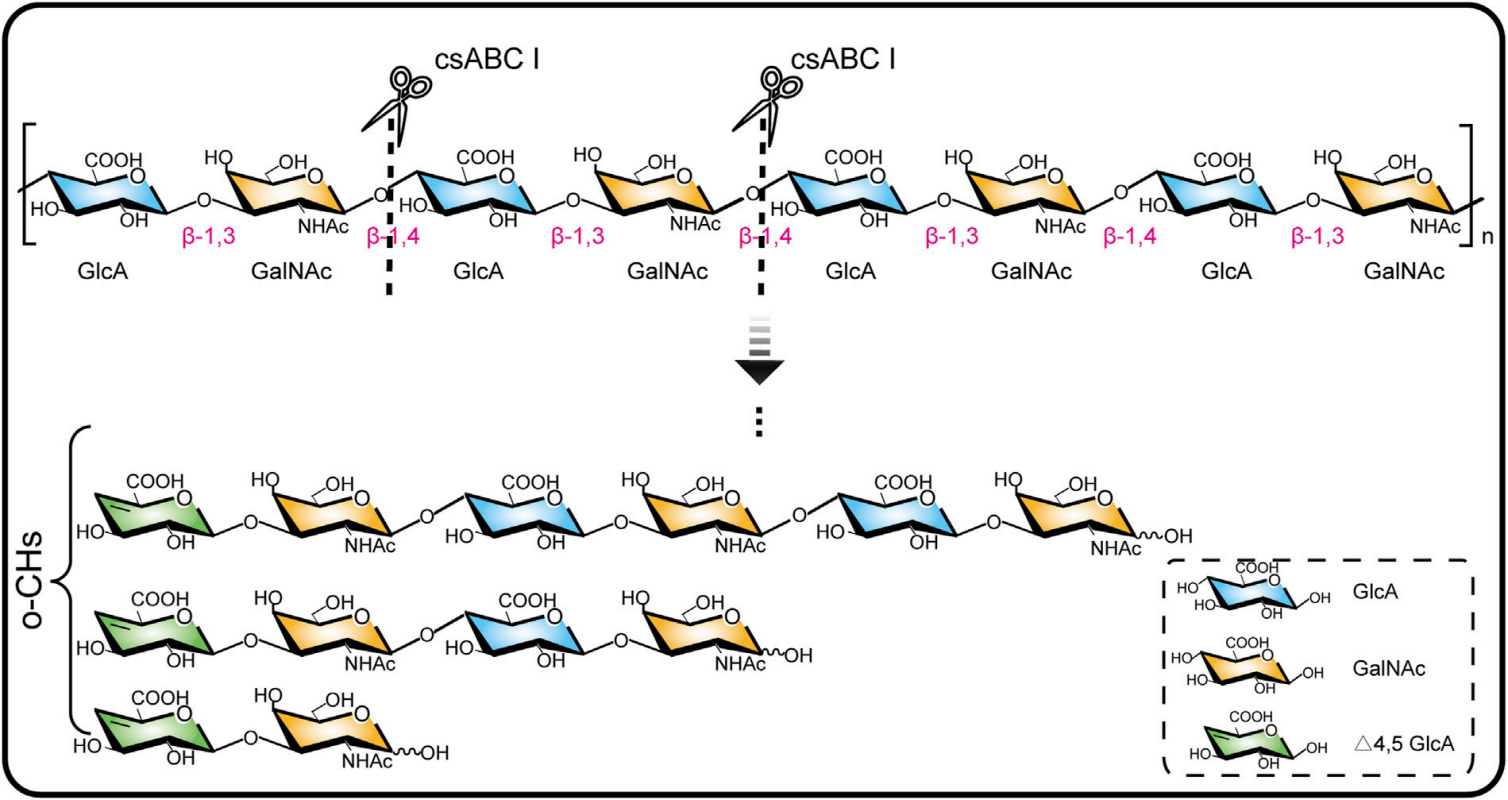
Chondroitinase ABC I depolymerizes chondroitin chain by breaking the β-1,4 glycoside bond.

**FIGURE 2:**
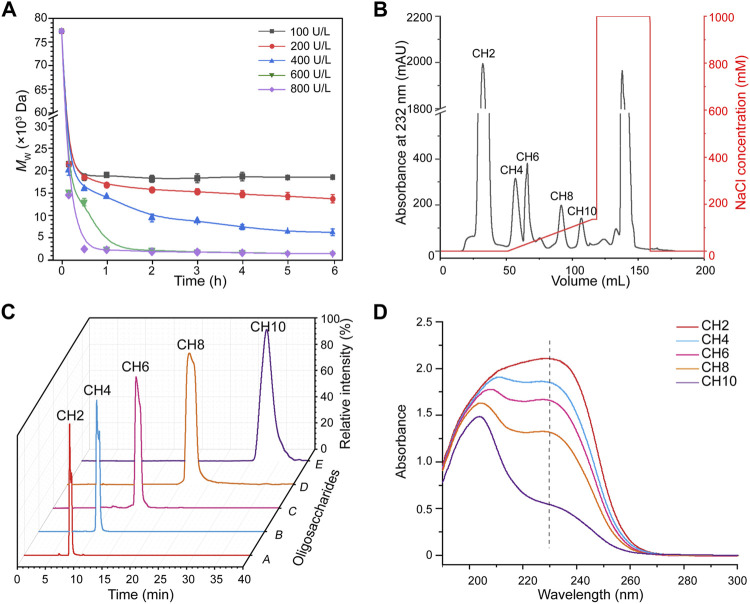
Production and characterization of chondroitin oligosaccharides. **(A)** Time course of chondroitin depolymerization with different concentrations of csABC I. **(B)** Fraction of chondroitin oligosaccharides were linearly eluted with 0–140 mM NaCl from a Q HP column at a flow rate of 3 ml/min. **(C)** Purity analysis of the CH2, CH4, CH6, CH8, and CH10 *via* HPLC. **(D)** Ultraviolet absorption spectrum of chondroitin oligosaccharides. *M*_w_, molecular weight; CH2, disaccharide; CH4, tetrasaccharide; CH6, hexasaccharide; CH8, octasaccharide; CH10, decasaccharide. The data are expressed as the mean ± SD from three (*n* = 3) biologically independent replicates.

csABC I as a polysaccharide lyase breaks the β-1,4 glycoside bonds in CS, which unique endolytic mode results in the generation of oligosaccharides with unsaturated GlcA at the non-reducing ends (Wang et al., 2019). Here we adopted a csABC I variant NΔ5/E694P (Wang et al., 2020) for depolymerizing *B. subtilis* sourced chondroitin (Zhou et al., 2018) (Figure 1). Our aim is to degrade chondroitin and isolate o-CHs with specific size since there has been no commercial oligosaccharides as standard samples. To this end, 15.0 g/L chondroitin was depolymerized by csABC I NΔ5/E694P with five different enzyme concentrations. Specifically, this protein csABC I NΔ5/E694P was purified from recombinant *E. coli* with a specific enzyme activity of 57.0 U/mg (Supplementary Figure S1) and the apparent *M*_w_ of chondroitin was 77.5 kDa (Supplementary Figure S2). As shown in Figure 2A, the apparent *M*_w_ decreased sharply under the depolymerization of csABC I in the first 1 h. Application of higher concentration of enzyme accelerated the depolymerization rate as well as decreased the apparent *M*_w_ of the final products (18,000, 14,000, 6,000, 1,300, and 1,200 Da, respectively), which was consistent with the results from CS (Wang et al., 2020). More recently, an *in vitro* method for synthesizing chondroitin fragments with cheap monosaccharides instead of UDP-GlcA and UDP-GalNAc was developed (Yang et al., 2021). Nevertheless, the established enzymatic approach here for producing low *M*_w_ chondroitin is more competitive since the bulk supply of chondroitin raw materials and controllable operation process.

As high enzyme activity tends to generate oligosaccharides with smaller size and vice versa, an intermediate enzymatic activity of 400 U/L was applied to generate more diversified oligosaccharides. As expected, the peaks corresponding to chondroitin disaccharides (CH2), tetrasaccharides (CH4), hexasaccharides (CH6), octasaccharides (CH8) and decasaccharides (CH10) were documented (Supplementary Figure S3). The molecular formula, molecular weight, negative ion masses and structure of all oligosaccharides were summarized in Supplementary Table S1. Moreover, the results also showed that o-CHs with the same molecular mass presented as two peaks (Supplementary Figure S3), which confirmed the oberved mutarotation between the α- and β-anomers of GalNAc as the reducing termini (Paul et al., 1987; Skelley and Mathies, 2006). On this basis, ion exchange chromatography (Davies et al., 2008; Shastri et al., 2013; Rothenhofer et al., 2015) was applied to separate size-specific o-CHs (Figure 2B). All the oligosaccharides CH2, CH4, CH6, CH8 and CH10 in products were separated (Supplementary Figure S4A) and identified with UPLC-MS. The [M-H]^−^ values of CH2, CH4, CH6, and CH8 were *m/z* 378.10, m/z 757.21, *m/z* 1136.33 and *m/z* 1515.44, respectively, while CH10 showed ion spectrum at *m/z* 946.76 ([M-2H]^2−^) (Supplementary Figure S4B). Further secondary ion mass spectrometry analysis confirmed the [M-H]^−^ value (*m/z* 1894.53) and other characteristic fragment ions of CH10 (Supplementary Figure S5).

After desalination and lyophilization, the purities of all the o-CHs (CH2, CH4, CH6, CH8 and CH10) were further analyzed by HPLC (Figure 2C). We also studied the ultraviolet absorption spectrum of these novel products. As shown in Figures 2A,D characteristic absorption wavelength near 232 nm was detected, which could be ascribed to the introduction of unsaturated bond during degradation. Besides, no absorption peaks at 260 and 280 nm were recorded, suggesting the absence of nucleic acids and proteins in the prepared standard samples (CH2, CH4, CH6, CH8, and CH10) and their structures were determined by ^1^H NMR (Supplementary Figures S6–S10). The production of o-CHs with specific size could be applied to study on structure-activity relationships and explore new biological functions.

### Quantitative Analysis of Depolymerization Towards Size-specific o-CHs

**FIGURE 3:**
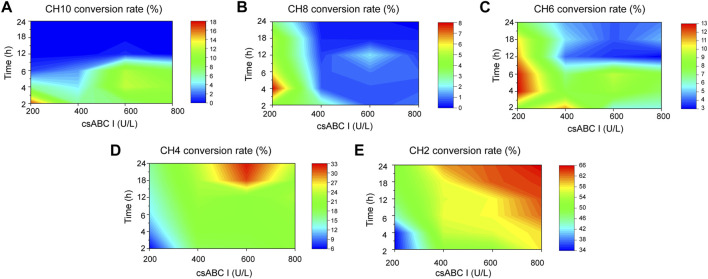
Orthogonalizing csABC I activities and depolymerization time to maximized the yield of o-CHs. **(A)** CH10, **(B)** CH8, **(C)** CH6, **(D)** CH4, **(E)** CH2. The csABC I activity and reaction time spanned 200–800 U/L and 2–24 h, respectively. CH2, disaccharide; CH4, tetrasaccharide; CH6, hexasaccharide; CH8, octasaccharide; CH10, decasaccharide. The data are expressed from three (*n* = 3) biologically independent replicates.

After preparation of standard samples, the standard curve of each oligosaccharide established by standards made in-house and shown in Supplementary Table S2. On this basis the depolymerization process was quantitatively analyzed to determine the best concentration of lyase and digestion time towards oligosaccharides with specific size. As shown in Figure 3A, the conversion rate of CH10 decreased with time under low enzyme activity, and reached a maximum value of 16.3% with 200 U/L csABC I NΔ5/E694P at 2 h. In contrast, with the same enzyme concentration, the maximum conversion rate of CH8 (7.2%) and CH6 (12.7%) occurred at 4 h (Figures 3B,C). These results suggested that low enzymatic activity and short depolymerization time were favorable to the production of CH10, CH8, and CH6. Moreover, the results also indicated that CH6 was an intermediate product and could be further depolymerized into smaller products CH4 and CH2.

As shown in Figure 3D, the conversion rate of CH4 raised gradually with the increasing supplement of enzyme activities in the range of 200–600 U/L. The highest conversion of CH4 reached 32.1% when depolymerized for 24 h with the enzyme activity of 600 U/L. However, when enzyme activity exceeding 600 U/L, the conversion rate of CH4 decreased, thus presumably CH4 should be the minimal depolymerizing substrate. This speculation was confirmed by csABC I cleavage of CH4 and CH6, as shown in Supplementary Figure S11A, CH6 could be completely converted to CH4 and CH2. In addition, when CH4 was depolymerized in 600 U/L csABC I for 12 h, only 16.9% of CH4 was slowly degraded to CH2 (Supplementary Figure S11B). Meanwhile, as shown in Figure 3E, the conversion rate of CH2 continually increased with the increase of enzyme activity and degradation time, and eventually reached a highest value of 65.5%. By controlling the enzyme activity of csABC I and depolymerization time, size-specific oligosaccharides CH2, CH4, CH6, CH8, and CH10 could be dedicatedly produced.

### Construction and Optimization of a Novel Sulfation System From Triphosphate and Sulfate

**FIGURE 4:**
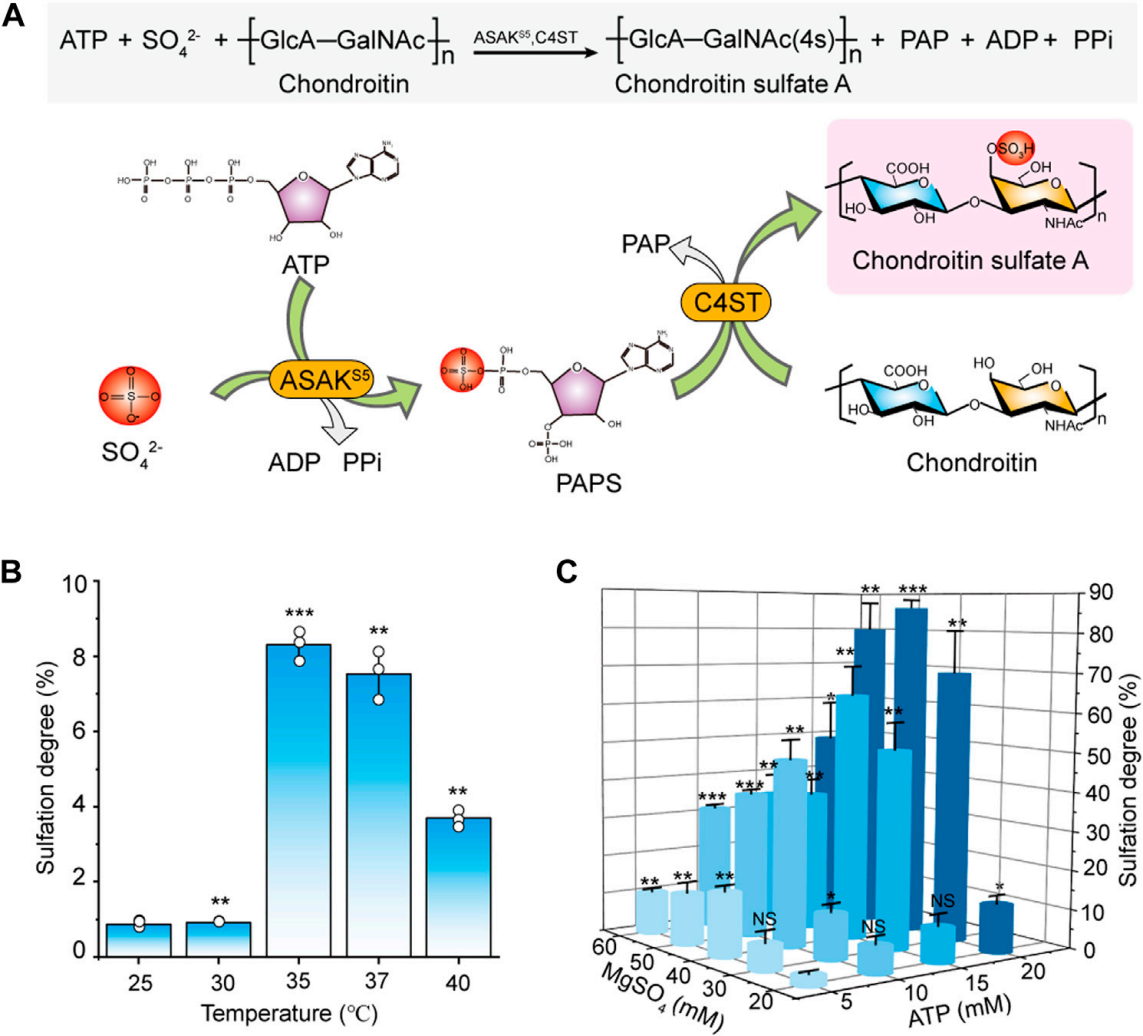
Construction and optimization of the sulfation system from ATP and sulfate. **(A)** Schematic diagram of sulfation-modification system for CSA biosynthesis. **(B)** The effect of temperature on sulfation in the reaction system with 10 mM ATP, 20 mM MgSO_4_, 0.5 g/L ASAK^S5^, 1.0 g/L C4ST, and 2.0 g/L chondroitin for 48 h. **(C)** The effect of ATP concentration and MgSO_4_ concentration on the sulfation in the system with ASAK^S5^ (0.5 g/L), C4ST (1.0 g/L) and chondroitin (2.0 g/L) at a reaction temperature of 35°C for 48 h. ATP, adenosine 5′-triphosphate; ADP, adenosine 5′-diphosphate; PAPS, 3′-phosphoadenosine-5′-phosphosulfate; PPi, pyrophosphate; ASAK^S5^, a bifunctional PAPS synthase that converts ATP to PAPS. The data are expressed as the mean ± SD from three (*n* = 3) biologically independent replicates. The statistical analysis was performed by two-sided *t*-test. *p < 0.05, **p < 0.01, ***p < 0.001; NS not significant (*p* ≥ 0.05).

According to our recent study on PAPS biosynthesis (Xu et al., 2021), a new green sulfation system that directly from ATP and sulfate for CS biosynthesis was established (Figure 4A). C4ST and ASAK^S5^ were purified from recombinant *P. pastoris* (Jin et al., 2020) and *E. coli* (Xu et al., 2021) respectively according to our previous research (Supplementary Figures S12A,S12B). In consideration of the critical effect of temperature on enzyme reaction (Arcus & Mulholland, 2020), the temperature of the entire catalytic system with C4ST and ASAK^S5^ was explored. As shown in Figure 4B, in comparison, 35°C was the optimum temperature for *in vitro* sulfation reaction with a sulfation degree of 8.3%. In addition, the concentrations of ATP and MgSO_4_ were also orthogonally optimized. With the increase of ATP concentration, the sulfation level significantly increased (Figure 4C) which should be ascribed to the rapid generation of PAPS. The results showed that with the presence of 20 mM ATP and 40 mM MgSO_4_, the sulfation degree of chondroitin reached to 85.8% (Figure 4C). When further increased the concentration of MgSO_4_, the sulfation efficiency decreased, suggesting the inhibitory effect of high concentration of MgSO_4_ on entire catalytic system. Although the sulfation rate was comparatively lower than the ASST-dependent system (Jin et al., 2020), this enzymatic system was more attractive since the exclusion of high cost of the substrates GalNAc, UDP-GalNAc, UDP-GlcA, PAP, and PAPS (Supplementary Table S3, Sigma-Aldrich) and the generation of toxic by-product PNP.

### Enzymatic Synthesis of Oligosaccharides Sulfate Derivatives

**FIGURE 5:**
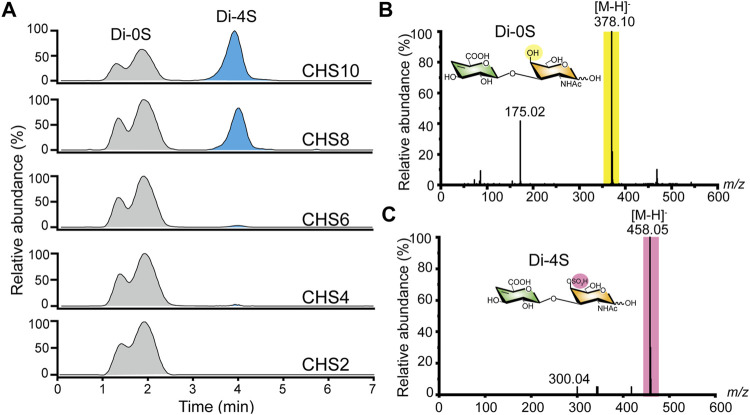
Characterization of chondroitin sulfate A oligosaccharides via UPLC-MS. **(A)** The ion chromatogram of Di-4S was determined by UPLC-MS. **(B)** MS spectra of chondroitin disaccharide (Di-0S) **(C)** MS spectra of chondroitin sulfate A disaccharide (Di-4S). Di-0S, chondroitin disaccharide; Di-4S, chondroitin sulfate A disaccharide; CHS2, chondroitin disaccharide sulfate derivative; CHS4, chondroitin tetrasaccharide sulfate derivative; CHS6, chondroitin hexasaccharide sulfate derivative; CHS8, chondroitin octasaccharide sulfate derivative; CHS10, chondroitin decasaccharide sulfate derivative.

**FIGURE 6:**
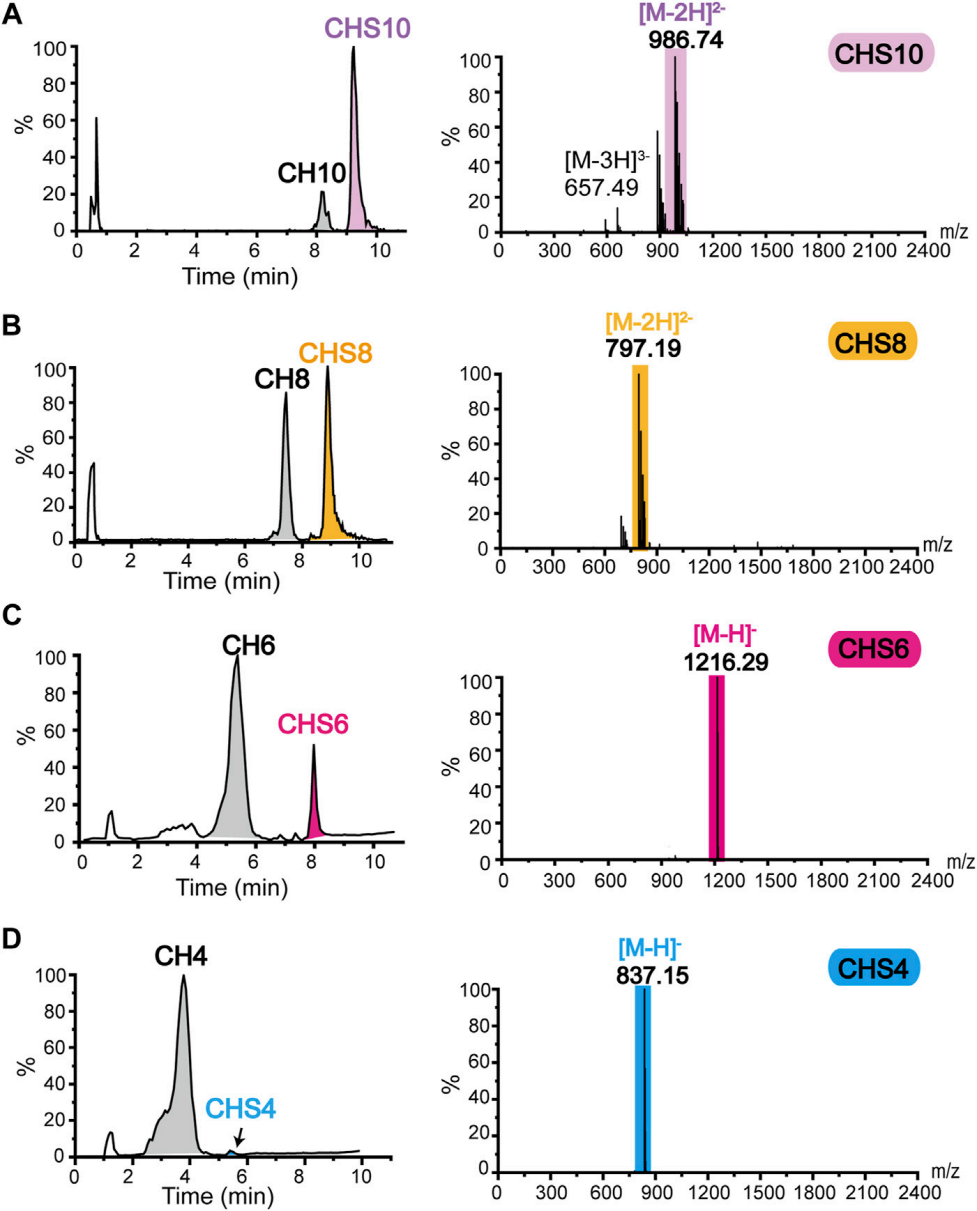
Mass spectrometry identification of sulfate derivatives of chondroitin oligosaccharides **(A)** Mass spectrometric identification of CH10 sulfated to CHS10. **(B)** Mass spectrometric identification of CH8 sulfated to CHS8. **(C)** Mass spectrometric identification of CH6 sulfated to CHS6. **(D)** Mass spectrometric identification of CH4 sulfated to CHS4. CHS2, chondroitin disaccharide sulfate derivative; CHS4, chondroitin tetrasaccharide sulfate derivative; CHS6, chondroitin hexasaccharide sulfate derivative; CHS8, chondroitin octasaccharide sulfate derivative; CHS10, chondroitin decasaccharide sulfate derivative.

After preparing size-specific o-CHs and constructing the ATP-dependent sulfation system, we concentrated on the enzymatic production of oligosaccharides sulfate derivatives. As shown in Figure 5A, the o-CHs were sulfated and then degraded by tool enzyme csABC, and all the Di-4S (CSA disaccharide) were analyzed by mass spectrometry. The sulfate derivatives of CH10, CH8, CH6, CH4, and CH2 were named CHS10, CHS8, CHS6, CHS4, and CHS2, respectively. According to the Di-4S peak, it could be found that CH10, CH8, CH6 and CH4 all could be sulfated except for CH2 (Figure 5A). This result demonstrated that tetrasaccharide was the smallest recognition unit for C4ST. Moreover, it could be found that the peak area of Di-4S increased with longer chains especially CH8 and CH10, which confirmed that C4ST has higher affinity towards o-CHs with long chains. The mass spectral peaks of CH2 *m/z* 378.10 and CSA disaccharide *m/z* 458.05 were also identified (Figure 5B). Additionally, all the sulfated oligosaccharides were further analyzed by UPLC-MS and the corresponding *m/z* values of CHS10 (Figure 6A), CHS8 (Figure 6B), CHS6 (Figure 6C) and CHS4 (Figure 6D) were documented in Supplementary Table S4, which were 986.74 ([M-2H]^2−^), 797.19 ([M-2H]^2−^), 1216.29 ([M-H]^−^), and 837.15 ([M-H]^−^), respectively. This result showed that only one sulfate group were added to o-CHs CH4, CH6, CH8, and CH10. To obtain CHS2, the enzymatically produced CS polysaccharides (Figure 4C) were depolymerized by csABC I. The oligosaccharides were separated and purified by an ion exchange column (Supplementary Figure S13A) and characterized by mass spectrometry (Supplementary Figure S13B).

## Conclusion

In this study, the enzymatic depolymerization process of chondroitin by chondroitinase ABC I was quantitatively analyzed and size-specific o-CHs CH2, CH4, CH6, CH8, and CH10 with defined structure were prepared with high conversion rates, which were 65.5%, 32.1%, 12.7%, 7.2%, and 16.3%, respectively. By optimizing the biosynthesis of PAPS from adenosine 5′-triphosphate and inorganic sulfate, a new green low-cost sulfation system was developed for chondroitin sulfation and the highest sulfation degree of chondroitin reached 85.8%. Eventually, artificial o-CHs sulfate derivatives including CHS10, CHS8, CHS6, CHS4, and CHS2 that with only one sulfate group were enzymatically produced. The present study provides new alternative approaches for the production of o-CHs and its sulfate derivatives, which would boost the studies of structure-activity relationship and broaden the applications of CS in medicine.

## Data Availability

The original contributions presented in the study are included in the article/Supplementary Material, further inquiries can be directed to the corresponding authors.
