# Supplementary material for: Enzymatic Production of Chondroitin Oligosaccharides and Its Sulfate Derivatives
Source: Front Bioeng Biotechnol. 2022 Jul 13;10:951740. doi: 10.3389/fbioe.2022.951740 (PMC9326237; doi:10.3389/fbioe.2022.951740)
Supplement: Supplementary file 1 [file DataSheet1.docx]

**Supplementary material**

**Enzymatic production of chondroitin** **oligosaccharides and its sulfate** **derivatives**

***Weijiao Zhang^a,b,c^, Ruirui Xu^a,b^, Xuerong Jin^a,b^, Yang Wang^a,b^, Litao Hu^a,b^, Tianmeng Zhang^a,b^, Guocheng Du^a,b*^, Zhen Kang^a,b*^***

*^a^The Key Laboratory of Carbohydrate Chemistry and Biotechnology, Ministry of Education, School of Biotechnology, Jiangnan University, Wuxi, China*

*^b^The Science Center for Future Foods, Jiangnan University, Wuxi, 214122, China*

*^c^The Key Laboratory of Industrial Biotechnology, Ministry of Education, School of Biotechnology, Jiangnan University, Wuxi, China*

*Corresponding authors: Zhen Kang, Guocheng Du

Phone: +86-510-510-85918307, Fax: +86-510-85918309

*E-mail address*: [zkang@jiangnan.edu.cn](mailto:zkang@jiangnan.edu.cn) or [gcdu@jiangnan.edu.cn](mailto:gcdu@jiangnan.edu.cn)

**Table S1** Information about the molecular formula, molecular weight, negative ion masses and structure of chondroitin oligosaccharides.

| Name | Oligosaccharides | Formula | Theor. *M*w | [M-H]^-^  (*m/z*) | Structures |
| --- | --- | --- | --- | --- | --- |
| CH2 | Disaccharide | C_14_H_21_NO_11_ | 379.11 | 378.10 | 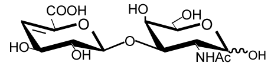 |
| CH4 | Tetrasaccharide | C_28_H_42_N_2_O_22_ | 758.22 | 757.21 | 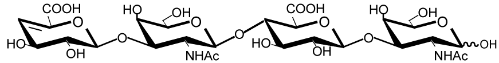 |
| CH6 | Hexasaccharide | C_42_H_63_N_3_O_33_ | 1137.33 | 1136.33 | 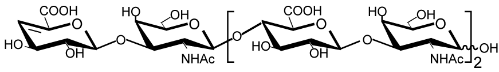 |
| CH8 | Octasaccharide | C_56_H_84_N_4_O_44_ | 1516.45 | 1515.44 | 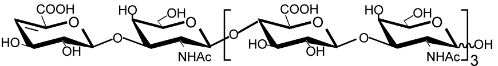 |
| CH10 | Decasaccharide | C_70_H_105_N_5_O_55_ | 1895.56 | 1894.53 | 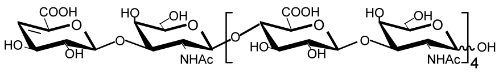 |

**Table S2** Summary of standard curves for o-CHs established by standards made in-house.

| Oligosaccharide | Regression equation | Correlation coefficient (R^2^) |
| --- | --- | --- |
| CH2 | y = 14154x - 1254.4 | 0.9976 |
| CH4 | y =7278.8x + 2410.5 | 0.9975 |
| CH6 | y = 5786.1x - 746.88 | 0.9975 |
| CH8 | y = 2715.8x - 62.411 | 0.9946 |
| CH10 | y = 1123.9x + 44.577 | 0.9961 |

x (g/L), concentration of o-CHs; y, the peak areas of o-CHs.

**Table S3** Selling price of different substrates on Sigma-Aldrich.

| Substrate | Cost ($/mg) |
| --- | --- |
| PAPS | 253.07 |
| UDP-GalNAc | 88.11 |
| UDP-GlcA | 5.13 |
| PAP | 41.07 |
| GalNAc | 5.37 |
| ATP | 0.05 |

**Table S4** Parent ion, negative ion masses and molecular weight of chondroitin sulfate oligosaccharides.

| Oligosaccharide | CHS4 | CHS6 | CHS8 | CHS10 |
| --- | --- | --- | --- | --- |
| Parent ion | [M-H]^-^ | [M-H]^-^ | [M-2H]^2-^ | [M-2H]^2-^ |
| *m/z* | 837.15 | 1216.29 | 797.19 | 986.74 |
| Molecular weight | 838.17 | 1217.28 | 1596.39 | 1975.51 |

**Figure S1**


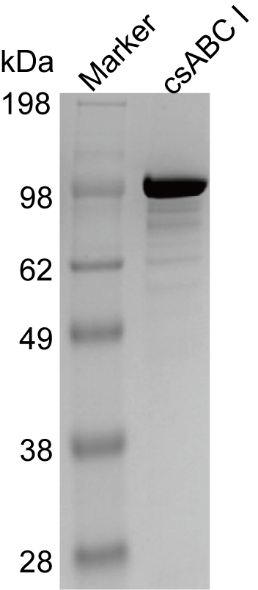


**Figure S1.** Expression of csABC I was analysed by SDS-PAGE.

**Figure S2**


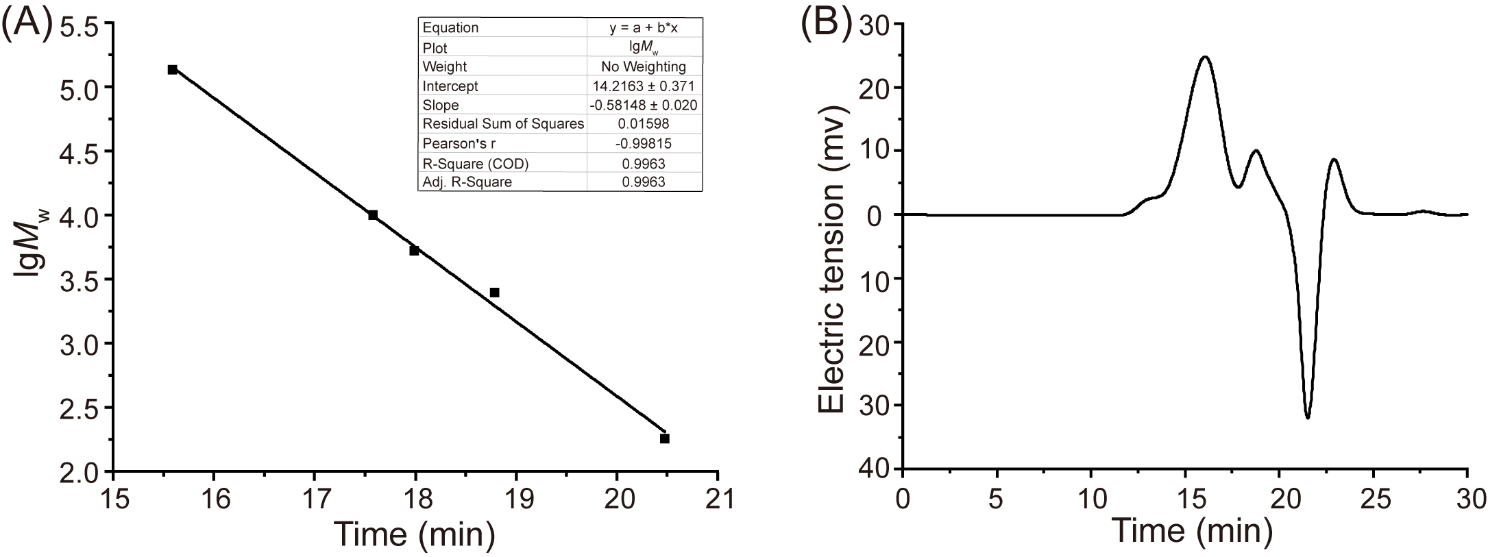


**Figure S2.** The apparent molecular weight of chondroitin determined by GPC-HPLC. (A) The calibration curve was obtained using different dextran molecular weights (180 Da, 2500 Da, 5250 Da, 10000 Da and 135350 Da). (B) GPC chromatogram of chondroitin.

**Figure S3**


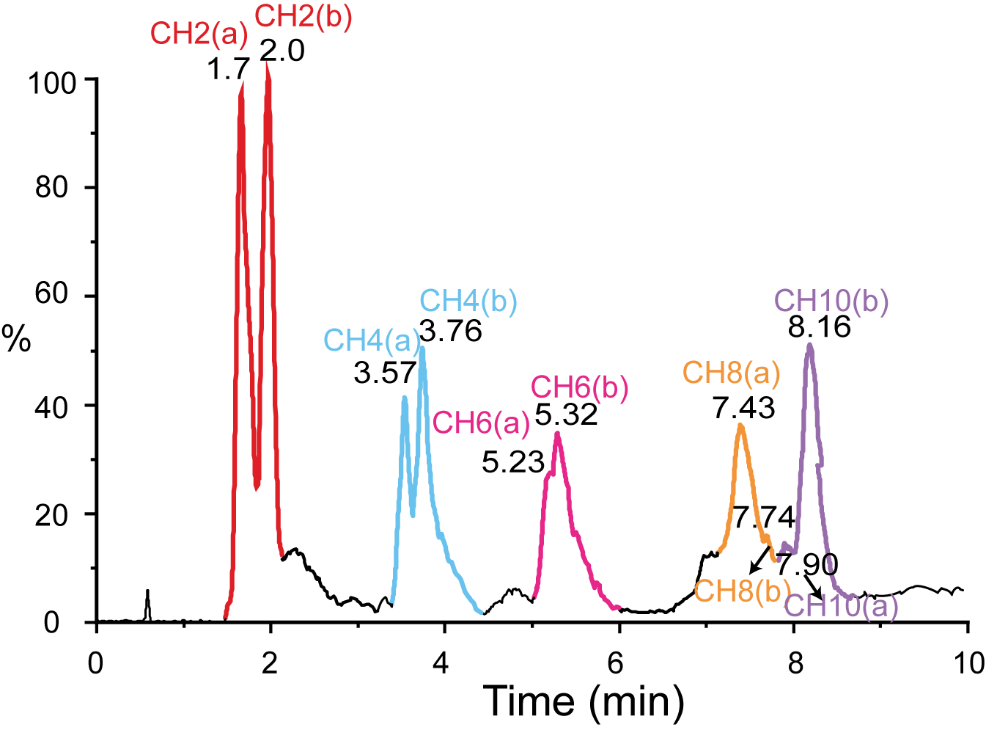


Figure S3. Ion chromatography of chondroitin mixture depolymerized at 400 U/L csABC I concentration for 2 h by UPLC-MS.

**Figure S4**


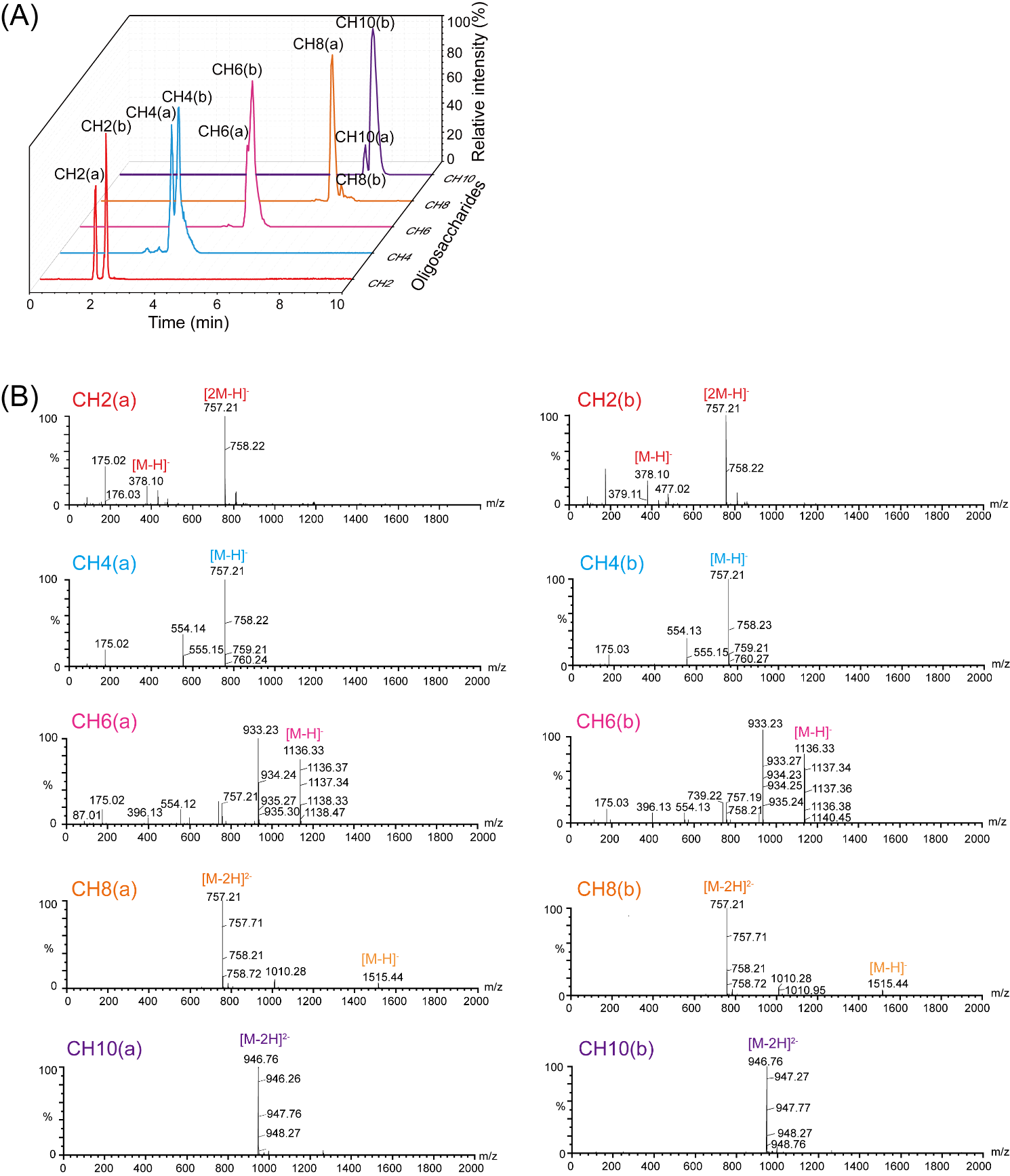


**Figure S4.** Pure o-CHs performed by UPLC-MS. (A) Ion chromatograms of pure o-CHs performed with an CSH C18 column. (B) MS spectra of o-CHs depolymerized at 400 U/L csABC I concentration for 2 h by UPLC-MS.

**Figure S5**


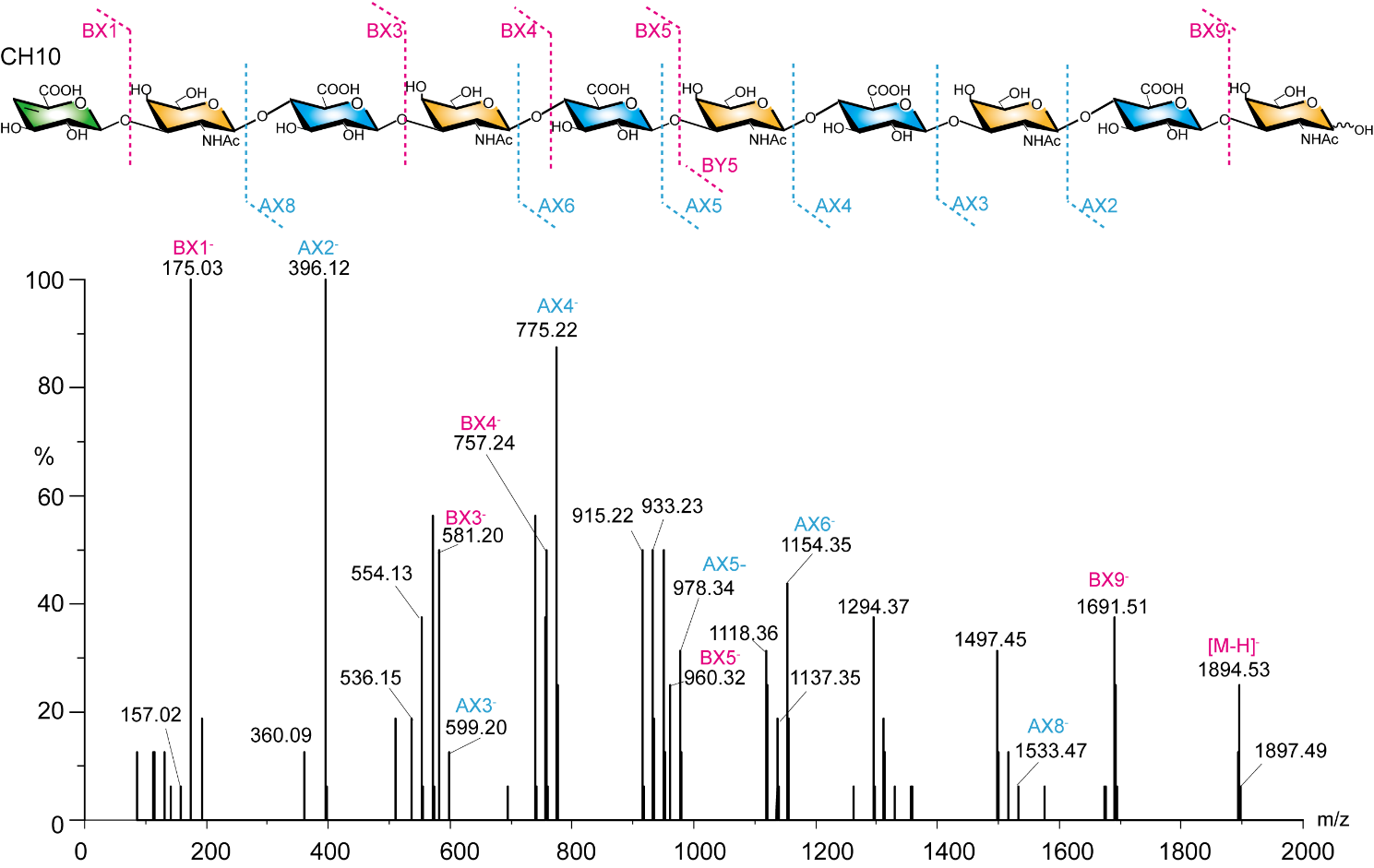


**Figure S5.** UPLC-MS/MS analysis of CH10.

**Figure S6**


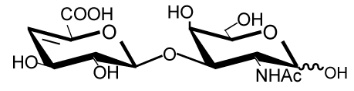


^1^H NMR (600 MHz, Deuterium Oxide) δ 5.94 (d, J = 4.1 Hz, 2H), 5.28 (d, J = 4.4 Hz, 2H), 5.24 (dd, J = 8.4, 4.0 Hz, 2H), 4.75 (d, J = 8.4 Hz, 2H), 4.32 (dd, J = 11.1, 3.8 Hz, 1H), 4.22 – 4.16 (m, 2H), 4.15 – 4.09 (m, 4H), 4.04 – 3.93 (m, 2H), 3.85 (s, 2H), 3.81 – 3.65 (m, 6H), 2.09 (s, 6H)


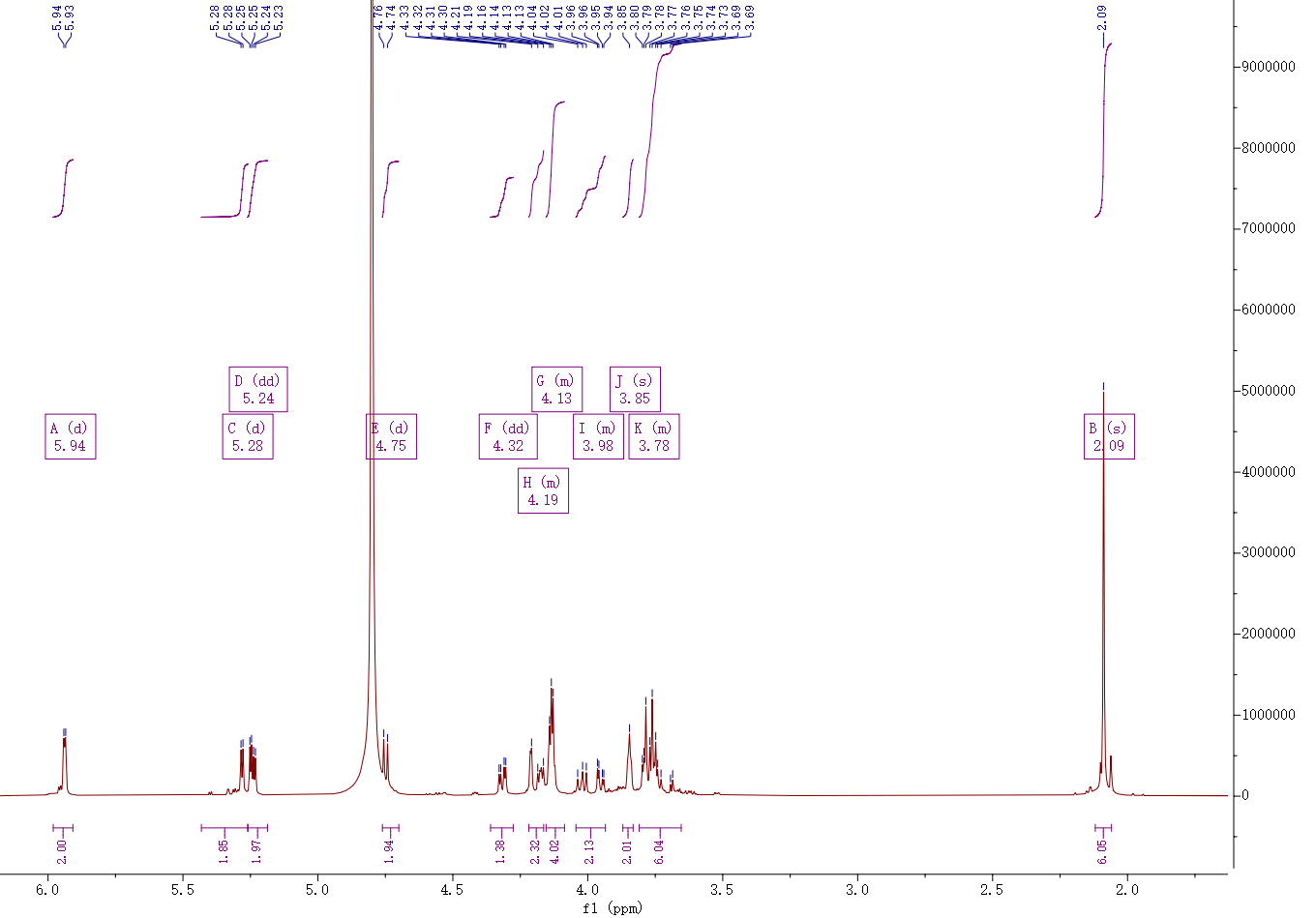


**Figure S6.** ^1^H-NMR spectra of CH2.

**Figure S7**


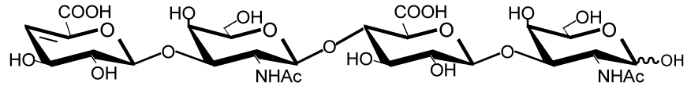


^1^H NMR (600 MHz, Deuterium Oxide) δ 5.92 (d, *J* = 4.1 Hz, 2H), 5.24 (d, *J* = 3.8 Hz, 2H), 5.21 (d, *J* = 4.4 Hz, 2H), 4.70 (d, *J* = 8.6 Hz, 2H), 4.61 – 4.50 (m, 4H), 4.39 – 4.20 (m, 4H), 4.12 (t, *J* = 4.0 Hz, 6H), 4.02 (q, *J* = 9.9 Hz, 4H), 3.93 (d, *J* = 10.9 Hz, 2H), 3.87 – 3.67 (m, 18H), 3.61 (td, *J* = 9.1, 4.9 Hz, 2H), 3.40 (t, *J* = 8.7 Hz, 2H), 2.07 (d, *J* = 22.4 Hz, 12H)


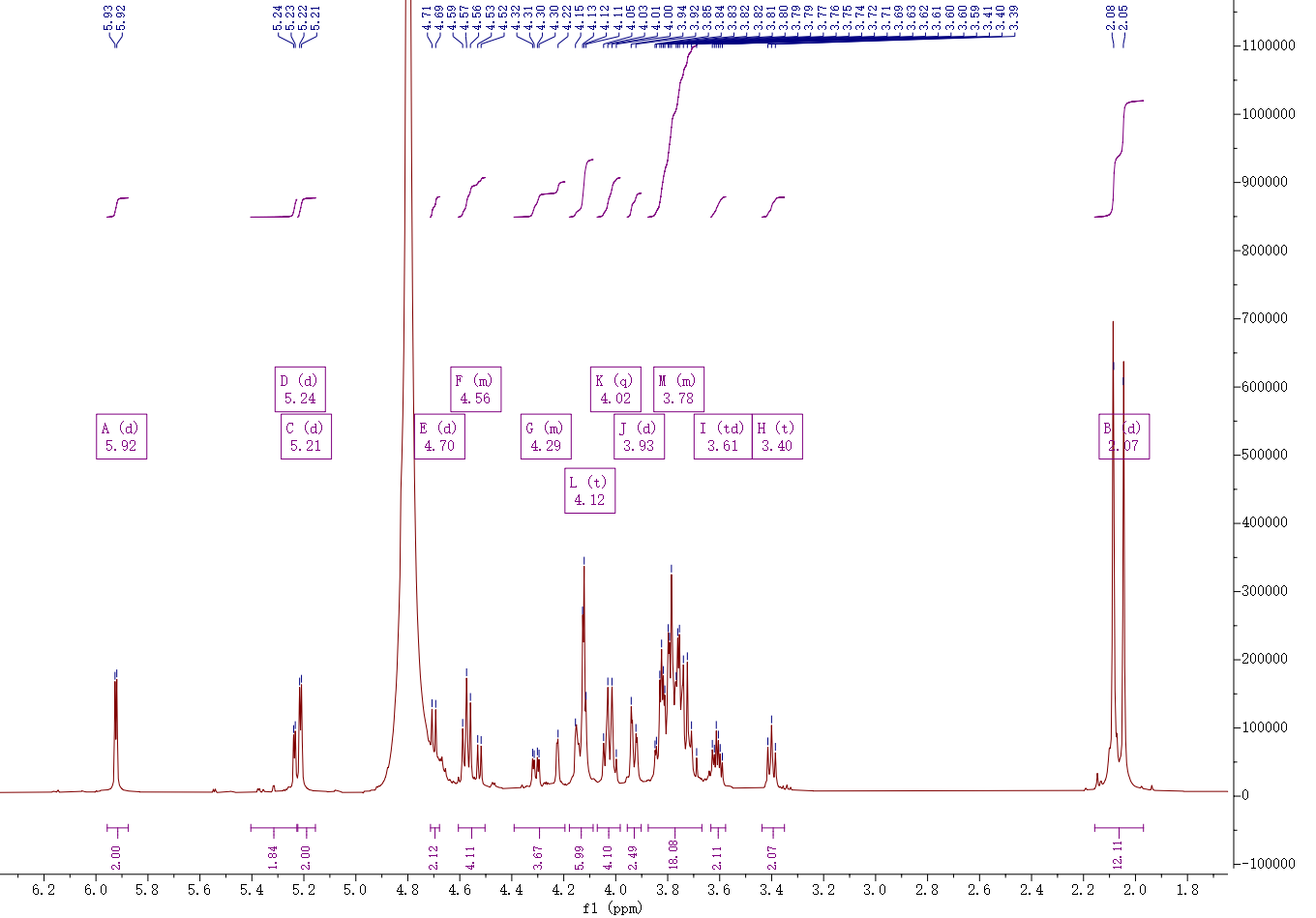


**Figure S7.** ^1^H-NMR spectra of CH4.

**Figure S8**


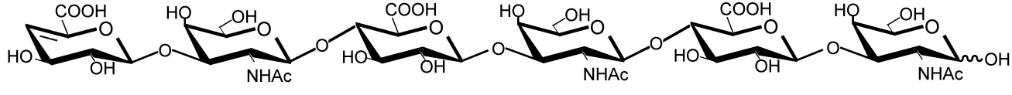


^1^H NMR (600 MHz, Deuterium Oxide) δ 5.92 (d, *J* = 4.1 Hz, 2H), 5.23 (d, *J* = 3.7 Hz, 2H), 5.21 (d, *J* = 4.1 Hz, 2H), 4.71 – 4.66 (m, 4H), 4.54 (dt, *J* = 29.6, 9.5 Hz, 8H), 4.36 – 4.20 (m, 10H), 4.13 (d, *J* = 16.1 Hz, 8H), 4.06 – 4.00 (m, 6H), 3.93 (d, *J* = 12.2 Hz, 4H), 3.87 – 3.69 (m, 30H), 3.61 (q, *J* = 8.4 Hz, 8H), 3.39 (q, *J* = 9.3 Hz, 6H), 2.06 (d, *J* = 23.1 Hz, 18H)


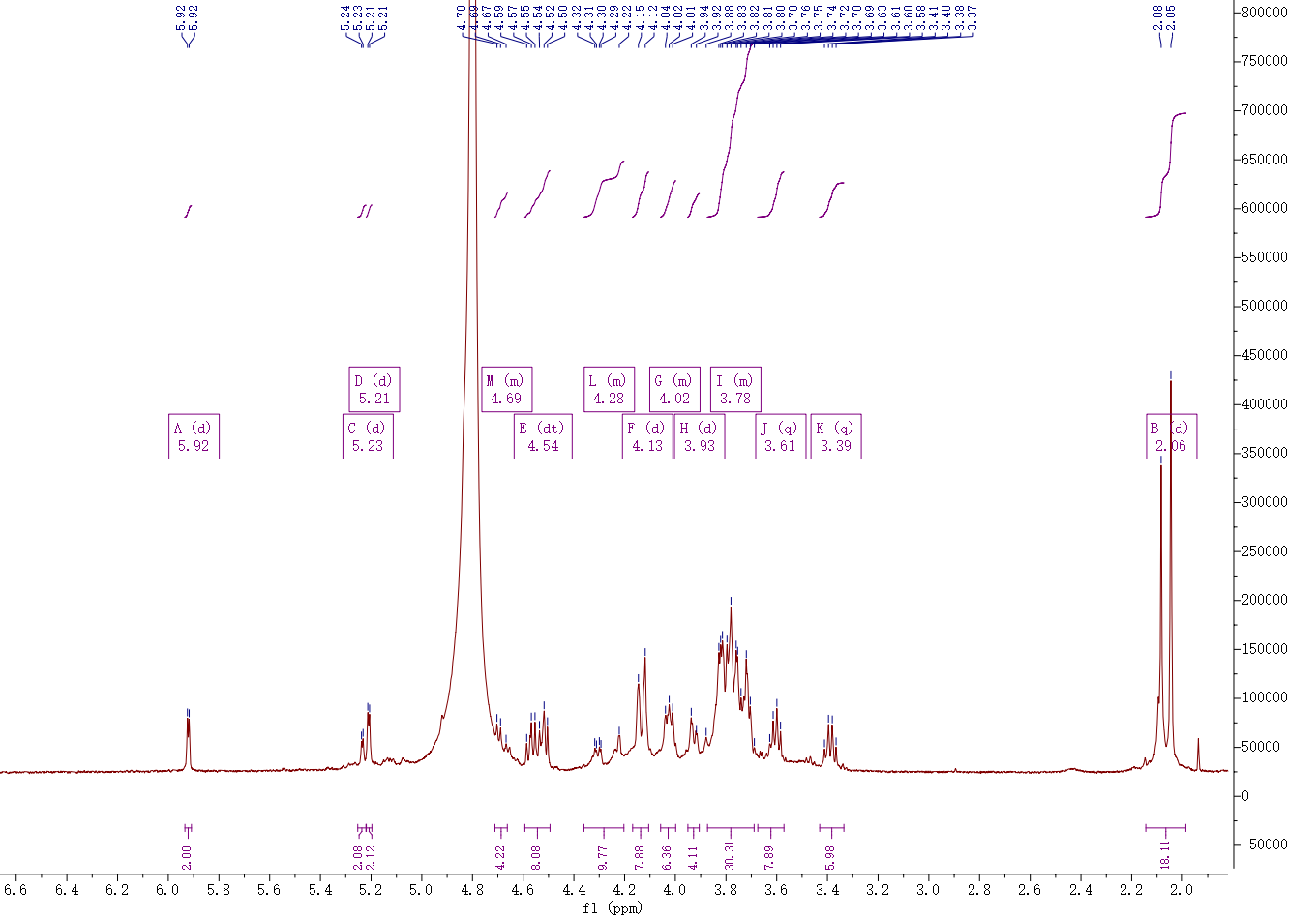


**Figure S8.** ^1^H-NMR spectra of CH6.

**Figure S9**


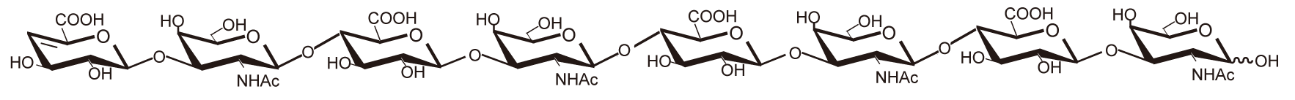


^1^H NMR (600 MHz, Deuterium Oxide) δ 5.92 (d, *J* = 4.2 Hz, 2H), 5.23 (d, *J* = 3.9 Hz, 2H), 5.21 (d, *J* = 4.3 Hz, 2H), 4.72 – 4.63 (m, 4H), 4.61 – 4.45 (m, 10H), 4.30 (dd, *J* = 10.9, 3.9 Hz, 2H), 4.22 (s, 2H), 4.16 – 4.09 (m, 8H), 4.02 (dd, *J* = 11.4, 3.6 Hz, 6H), 3.97 – 3.89 (m, 4H), 3.88 – 3.67 (m, 36H), 3.60 (q, *J* = 8.5 Hz, 6H), 3.38 (q, *J* = 10.0, 9.0 Hz, 6H), 2.06 (d, *J* = 23.6 Hz, 24H)


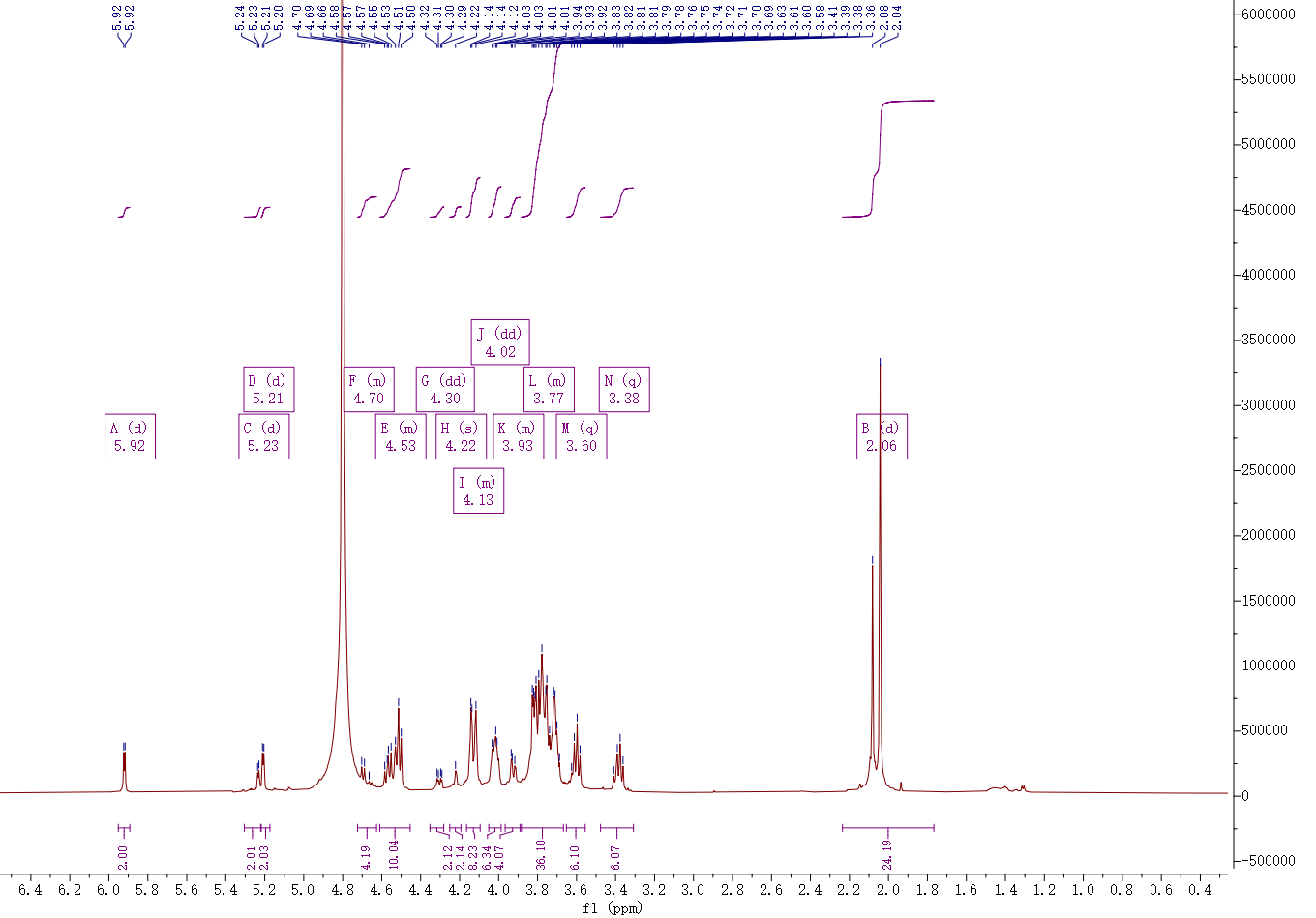


**Figure S9.** ^1^H-NMR spectra of CH8.

**Figure S10**


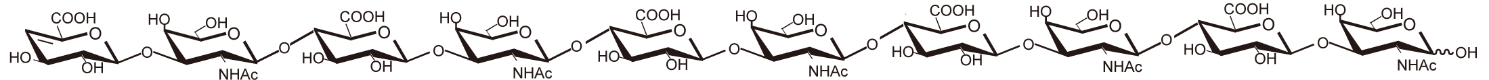


^1^H NMR (600 MHz, Deuterium Oxide) δ 5.92 (d, *J* = 4.1 Hz, 2H), 5.23 (d, *J* = 3.9 Hz, 2H), 5.21 (d, *J* = 4.3 Hz, 2H), 4.69 (d, *J* = 8.4 Hz, 2H), 4.59 – 4.55 (m, 4H), 4.51 (t, *J* = 9.0 Hz, 8H), 4.30 (dd, *J* = 11.0, 3.7 Hz, 2H), 4.22 (s, 2H), 4.13 (d, *J* = 13.6 Hz, 10H), 4.01 (t, *J* = 9.8 Hz, 10H), 3.92 (d, *J* = 10.9 Hz, 4H), 3.87 – 3.65 (m, 40H), 3.60 (t, *J* = 8.9 Hz, 8H), 3.38 (q, *J* = 10.4, 8.7 Hz, 8H), 2.06 (d, *J* = 23.7 Hz, 30H)


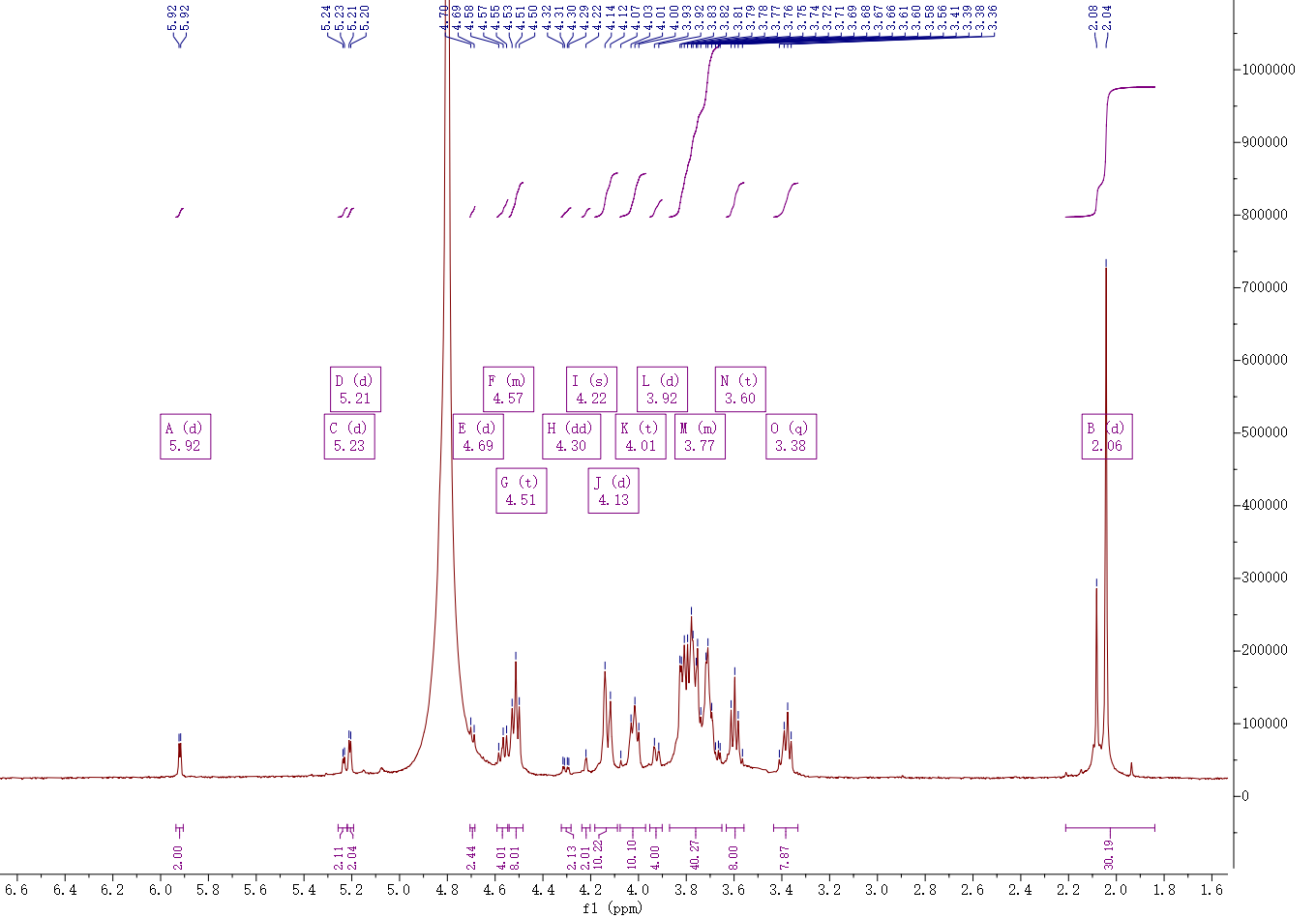


**Figure S10.** ^1^H-NMR spectra of CH10.

**Figure S11**


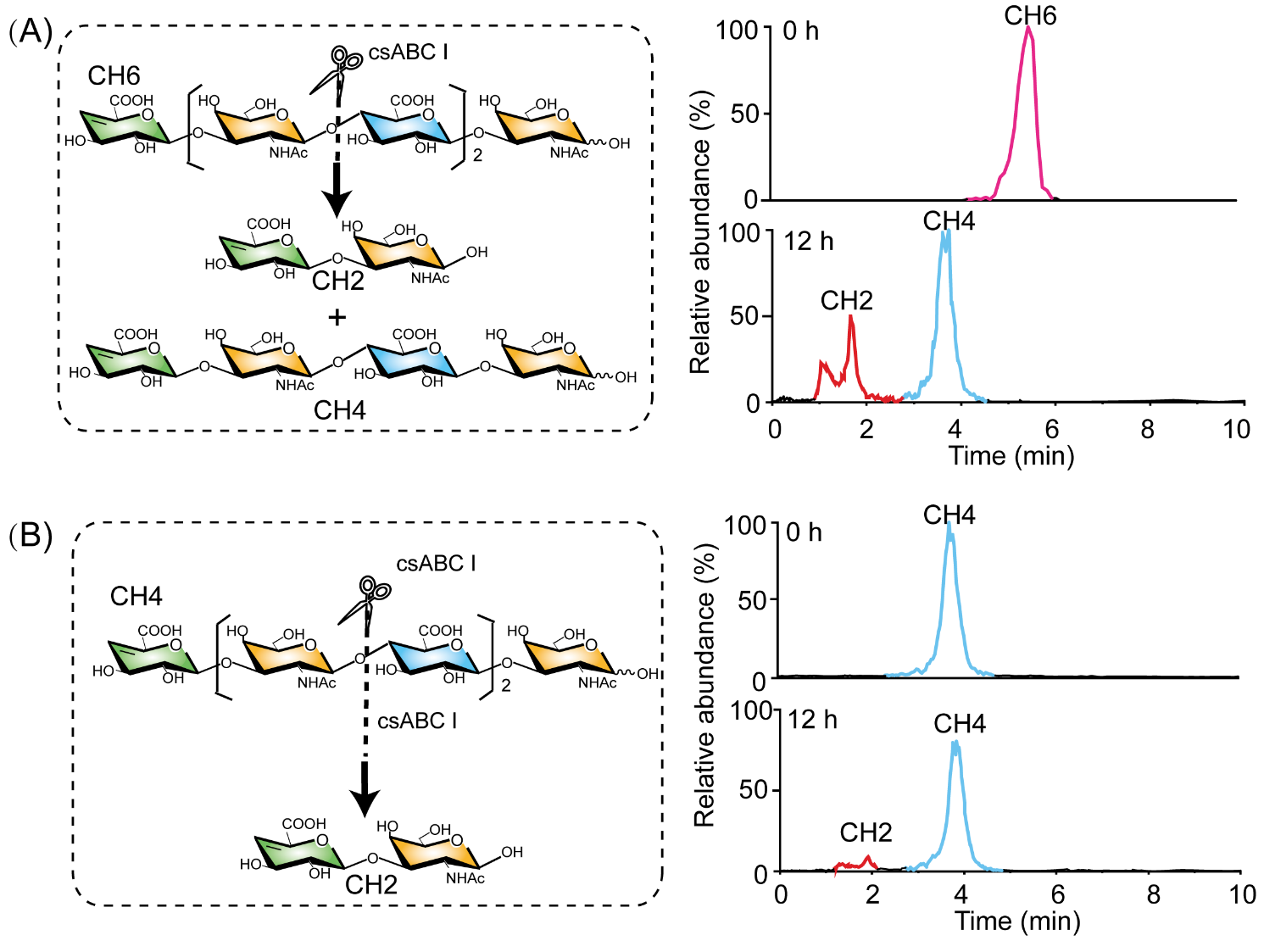


**Figure S11.** Digestion of chondroitin tetrasaccharide and chondroitin hexasaccharide with csABC I. (A) Chromatograms of digested oligosaccharide products from CH6 at 0 h and 12 h. (B) Chromatograms of digested oligosaccharide products from CH4 at 0 h and 12 h.

**Figure S12**


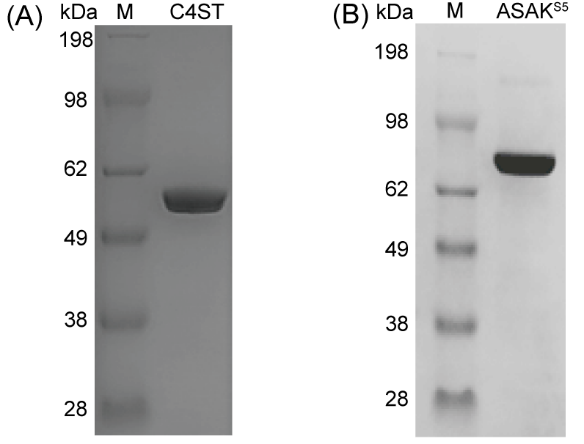


**Figure S12.** Expression of C4ST and ASAK^S5^ were analysed by SDS-PAGE.

**Figure S13**


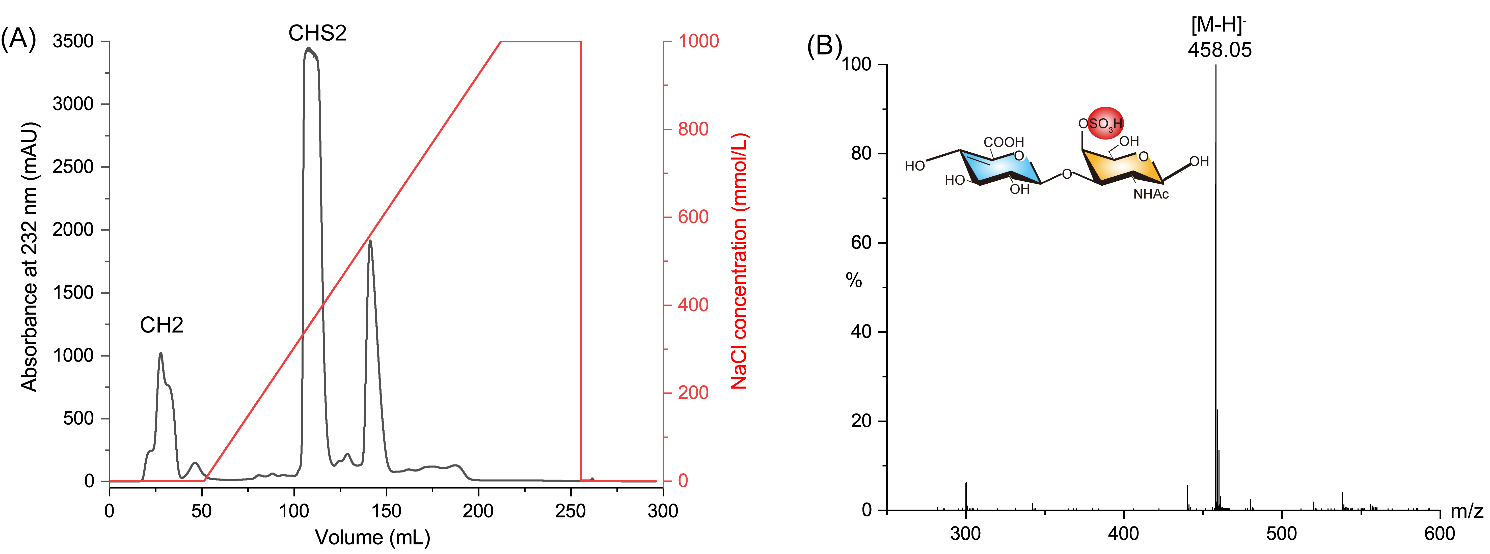


**Figure S13.** Depolymerization analysis of CS by csABC I. (A) Anion-exchange chromatograms of depolymerization products linearly eluted with 0–1000 mmol/L NaCl on a Q HP column at a flow rate of 3 mL/min. (B) MS spectra of CHS2.
